# Biosensors Designed for Clinical Applications

**DOI:** 10.3390/biomedicines9070702

**Published:** 2021-06-22

**Authors:** James F. Rusling, Robert J. Forster

**Affiliations:** 1 Department of Chemistry, University of Connecticut, Storrs, CT 06269, USA; 2 School of Chemistry, National University of Ireland, H 91 Galway, Ireland; 3 UConn Health Center, Farmington, CT 06032, USA; 4 Institute of Materials Science, University of Connecticut, Storrs, CT 06269, USA; 5 School of Chemical Sciences, Dublin City University, D9 Dublin, Ireland; robert.forster@dcu.ie; 6 FutureNeuro SFI Research Centre, D9 Dublin, Ireland

**Keywords:** electrochemical sensors, clinical analysis, biomarkers, electrochemiluminescence, cancer, cardiovascular and neurological diseases, epilepsy, microfluidics, point of care

## Abstract

Emerging and validated biomarkers promise to revolutionize clinical practice, shifting the emphasis away from the management of chronic disease towards prevention, early diagnosis and early intervention. The challenge of detecting these low abundance protein and nucleic acid biomarkers within the clinical context demands the development of highly sensitive, even single molecule, assays that are also capable of selectively measuring a small number of defined analytes in complex samples such as whole blood, interstitial fluid, saliva or urine. Success relies on significant innovations in nanomaterials, bioreceptor engineering, transduction strategies and microfluidics. Primarily using examples from our work, this article discusses some recent advance in the selective and sensitive detection of disease biomarkers, highlights key innovations in sensor materials and identifies issues and challenges that need to be carefully considered especially for researchers entering the field.

## 1. Introduction

Sensors are all around us and many were first devised long ago. For example, temperature sensors or “thermoscopes” were known in the time of Galileo, and the first modern thermometer, and accurate temperature sensor, was invented by D. Fahrenheit in 1709 [1]. Biosensors are sensors that detect or quantify analytes with biological or biomedical significance. Lyons and Clark reported the first glucose biosensor in 1962 which was probably the first usable biosensor and eventually led to widespread electrochemical glucose sensors [2] for home care [3,4]. Today, portable glucose sensors have revolutionized the care of diabetic patients moving the measurement of the blood glucose levels out of a centralized lab into the hands of the patients themselves. This strategy empowers the patient to take control of the management of their disease, allowing dramatically more frequent measurement as well as significantly improving patient outcomes and decreasing the overall cost of care [5].

To achieve current goals of point of care (POC) use, i.e., at the patient’s bedside, in a physician’s office or clinic, or by the patients themselves, the measurement protocol needs to be simple, foolproof, and usable by untrained personnel. Good examples include modern glucose sensors, where patients use test strips and a small device to draw nL blood samples and deliver them to the strip. The sample-charged strip is them inserted into a recalibrated meter that reads the glucose level and provides this information to the patient. In some cases, the protocol is more demanding and must be automated and packaged into a simple device to make it suitable for POC use. For example, immunosensors, which are widely used for measurements of proteins, antibodies, and many other types of biomolecules, often require wash steps and possible additional reagents. These challenges result in only a small fraction of recently published papers reporting automated or semiautomated protocols [6].

Patient prognosis can be significantly improved by quantifying appropriate biomarkers of disease (proteins, antibodies, nucleic acids and cells) that are found in abnormal quantities in body fluids or tissue when disease is present [7]. For example, microRNAs (miRNA) are short nucleotide sequences (typically 20–25 bases) that are present in the tissue and blood, plasma or serum of patients and show significant promise for early detection of disease, prognostic assessment, and monitoring of therapeutic efficacy [8]. One of the major challenges is that the concentration of miRNA is typically ultralow, i.e., picomolar or lower. Significant effort continues to be invested in increasing the signal associated with the binding of these biomarkers to a sensor capture surface. However, if these strategies also enhance the background response, the improvement in analytical performance with real world samples can fall short of what is needed. For example, increasing the surface area of an electrochemical sensor by using a nanostructured material increases the current for a redox probe in solution or the surface coverage of a labeled secondary antibody or nucleic acid probe strand, but will also increase the background charging current against which the faradaic current must be measured. The key challenge is to increase the signal to noise ratio. Approaches such as electrocatalysis and electrochemiluminescence [9,10] are particularly attractive in this context since the background current or light response can be made extraordinarily low [9]. In this approach, binding of the biomarker is followed by the probe (protein, secondary antibody or nucleic acid) that is labeled with an electrocatalyst, e.g., a metal complex or nanoparticle, or electrochemiluminescent label, e.g., luminol or ruthenium bpy complex, and triggers a large increase in the signal superimposed on a very low background.

Another major challenge is to selectively detect the disease biomarkers in the presence of tens of thousands of proteins and other nucleic acids that have the potential to interfere with the analysis. Selective detection requires careful attention to the performance of the bioreceptor itself, the way in which it is immobilized on the sensor surface and the transduction strategy, e.g., to minimize the response to nonspecifically bound components and maximize the signal arising from bound targets rather than those in solution.

In this paper, we describe several examples of biosensors, and their accompanying measurement protocols, that are aimed at clinical or POC use. These systems were designed for specific medical diagnostic application and employ automation, or at least semi-automation with an eye towards future POC use. We stress that this paper is not a general review of the field, but more of a tutorial on the development of working clinical methods involving measurements with novel biosensors. We are not attempting to cover a broad range of applications but focus on important design issues we have encountered in our own specific applications.

We consider cancer diagnostics by detecting cancer cells as well as protein biomarkers neurological diseases through microRNA profiling and cardiovascular disease through protein biomarker detection. Moreover, we reflect on the engineering and transduction innovations needed to deliver POC devices including microfluidics, 3D printing for rapid prototyping, and as well as electrochemical, chemiluminescent, and electrochemiluminescent detection methods. 

Since several of the following applications involve measuring biomarker proteins, we will introduce them here. The U. S. National Institutes of Health (NIH) defines biomarkers as “*molecules that can be reliably and accurately measured and are indicators of normal or disease biological processes and responses to therapeutic interventions*” [11]. Molecular biomarkers for diseases include nucleic acids, secreted proteins, antibodies, and small molecule metabolites. Measuring proteins in blood or cells provide a “snapshot” of the patient’s health [11]. For cancer diagnostics, serum protein measurements promise early detection and monitoring of therapy and post-surgery remission [12,13,14,15,16,17]. Cancer biomarker proteins have been discovered for every major cancer, and many are approved for use by the US FDA (Table 1).

Diagnostic proteins are secreted into the blood at higher or lower levels than normal when cancers begin to develop. The FDA-approved prostate specific antigen (PSA) is currently the only widely used clinical serum protein biomarker [19,20]. Biomarker proteins like PSA, are often elevated from inflammation and some may overexpress in several different types of cancers, i.e., they often lack specificity. Thus, single biomarker tests can be unreliable, and small panels of proteins give more accurate diagnostics than single biomarkers [11]. Current commercial assays for such panels are relatively expensive and sometimes lack the necessary sensitivity and few panels have been fully validated against known patient samples. For these reasons, their use in hospitals and clinics remains low.

**Figure 1:**
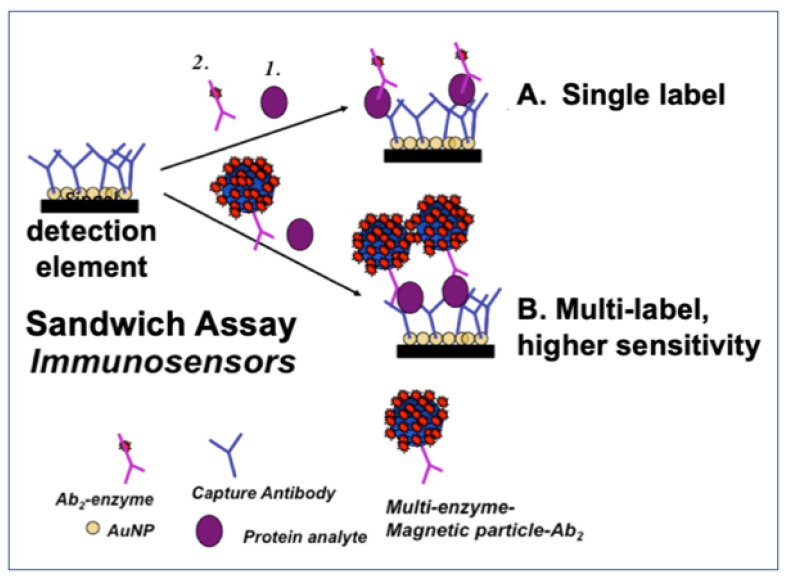
Principles of ELISA-like sandwich assays for proteins utilizing a capture antibody (Ab_1_) on a surface and a single or multiple labeled detection antibody (Ab_2_). Detection of labels can employ several techniques, including optical and electrochemical methods.

The time-honored protein detection method is the enzyme-linked immunosorbent assay (ELISA) [21]. Standard ELISA employs absorbance of a colored enzyme reaction product to achieve limits of detection (LOD) for proteins of 1–20 pg mL^−1^, with clinically relevant dynamic ranges. The method employs two antibodies in a “*sandwich assay*” of the analyte protein (Figure 1). Classic ELISA is limited by analysis time, sample size, cost, and lack of multiplexing. However, modern multiplexed commercial methods based on ELISA such as Luminex, Mesoscale and Quansys solve some of these problems [13]. The power of microfluidics was demonstrated in diagnostics more than a decade ago, in an example using sandwich assays with gold nanoparticle labels and silver deposition to detect antibodies against HIV and syphilis-causing agent [22]. Quanterix Simoa HD-X uses a microfluidic-based single protein counting detection using a sandwich assay and fluorescence detection with low fg/mL LODs [23], but the measuring instrument is very expensive.

In the following sections, we describe a series of projects from our own lab that were specifically designed for real clinical applications. Once again, this paper is not a general review article, but is written partly as a tutorial and includes discussion of problems that needed to be solved and how we addressed them. First, we discuss a low cost microfluidic immunoarray designed for detection of oral cancer by electrochemical detection of a four protein biomarker panel in serum, and that we also applied to prediction of oral cancer mucositis during drug/radiation treatments. Second, we discuss a more sophisticated microfluidic system that can predict whether prostate cancer patients need biopsies, and also a low cost 3D printed variant of this array that employs electrochemiluminescence (ECL) for detection [9]. Third, we describe a 3D printed immunoarray with a cell disruption unit designed to detect a metastatic oral cancer biomarker in cells. Fourth, we describe a label-free electrochemical impedance system to detect cancer cells. Fifth, we describe a microfluidic device designed to measure microRNAs in serum to monitor neurological disease. Finally, we consider recombinant antibody technology for the detection of the cardiac biomarker, C-reactive protein.

## 2. Important Optimization Issues

The first three examples discussed involve multiplexed measurement of protein cancer biomarkers with microfluidic immunoarrays, so we will begin by summarizing some of the general features of these systems that we have found to be essential for high sensitivity and low LODs. Nanostructured biosensors featuring single wall carbon nanotube (SWCNT) forests, AuNPs, or hydrogel networks decorated with capture antibodies combined with massive multilabel measurement strategies associated with the detection antibody can lead to high sensitivities with LODs in the single digit fg/mL range or lower. This feature is much more important than it seems, even if very low LODs are not necessary to detect the target protein analytes. This is because high sensitivity allows the clinical sample, e.g., plasma or even whole blood, to be diluted significantly which minimizes antibody cross reactivity due to low levels of protein analytes, decreases nonspecific binding (NSB) interferences, and can be traded off for faster assay times when target protein levels are well above the LOD [24].

In general, all reagent concentrations, flow rates, and stop-flow periods for incubations must be optimized to achieve the largest signal differences between protein levels in the desired linear concentration range and target free blanks. Minimizing nonspecific binding (NSB) of sample matrix molecules and labeled species on the biosensor surfaces is also very important. This is usually achieved by coating the biosensor surfaces with ~1% bovine serum albumin or casein or including nonionic detergents like ~0.05% Tween20 in the buffers and washing the entire microfluidic system with PBS/Tween20 solutions before assays. Appropriate levels of NSB minimization reagents should be tested and optimized for each new system. Optimizations should be done in the actual sample medium to be used or an appropriate surrogate. For human serum samples, we often use commercial calf serum as a surrogate since it contains similar protein levels as human serum, but no human proteins [25]. Therefore, if the human serum samples are to be diluted 100-fold by phosphate buffer saline (PBS), protein standards could be prepared in 1% calf serum in PBS. The most important optimizations in our hands have been the capture and detection antibodies, since use of the incorrect levels of antibodies in the assay can result in very small signals for samples, or even no signal at all above that of the blank. Antibodies should be monoclonal if possible, or one monoclonal and one polyclonal per protein, and have dissociation constants (K_D_) <10 nM with partner proteins.

## 3. Microfluidic Detection of Oral Cancer

**Figure 2:**
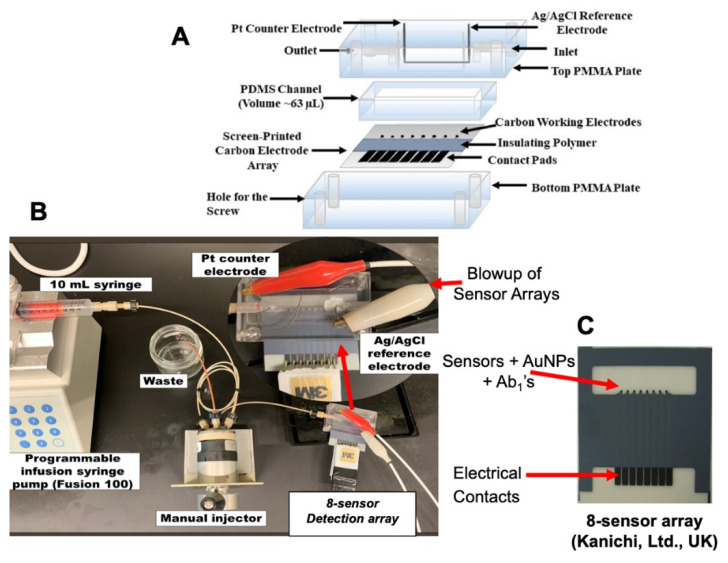
Electrochemical immunoarray for protein detection showing (**A**) exploded view of microfluidic detection channel featuring molded polydimethylsiloxane (PDMS) channel enclosing and 8-sensor array and Ag/AgCl reference and Pt-wire counter electrodes running the full length of the channel, (**B**) full microfluidic system showing programmable syringe pumps, injector, and 8-sensor array, and (**C**) Kanichi 8-carbon sensor array coated with Au-nanoparticle layers and attached capture antibodies.

**Figure 3:**
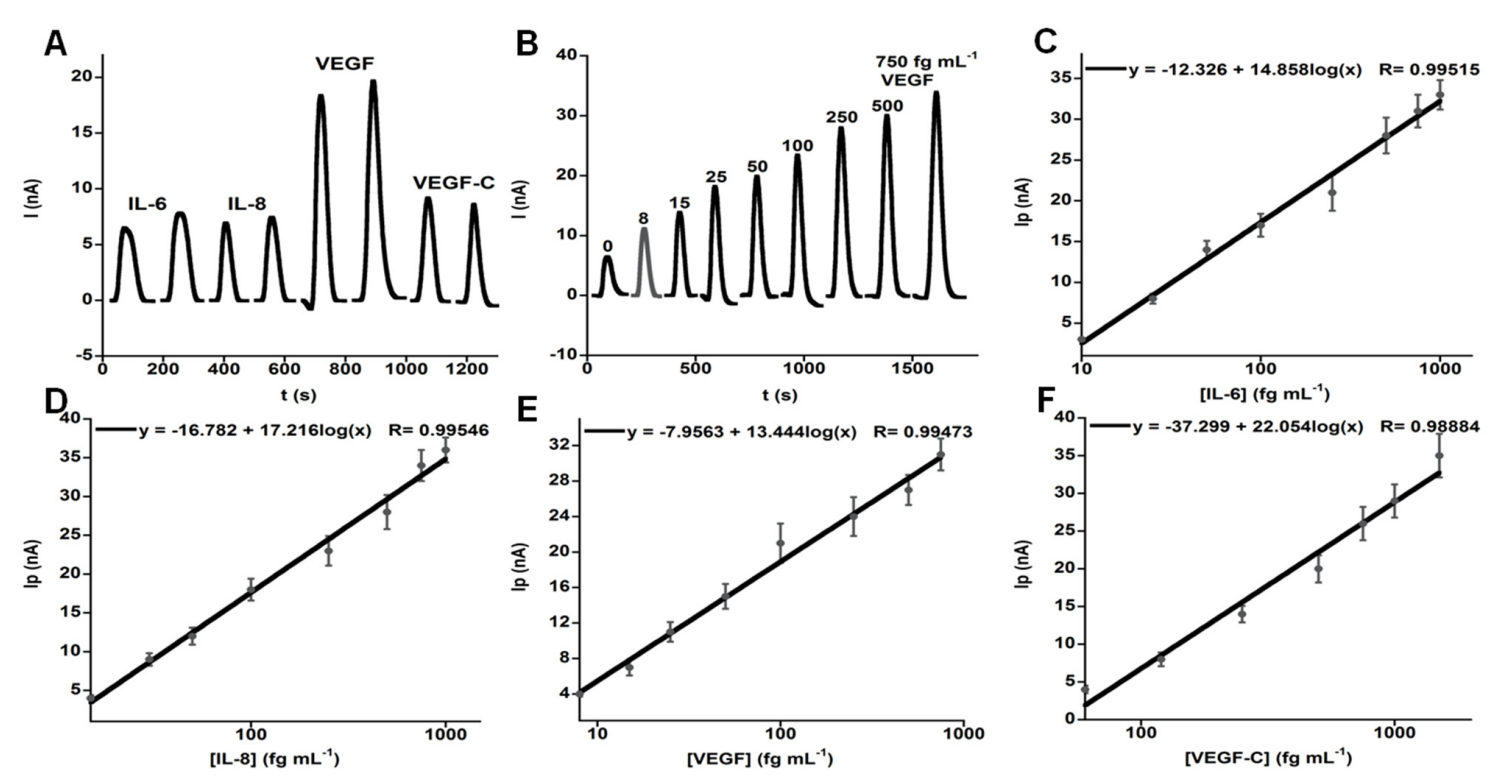
Oral cancer biomarker proteins detected in 1% calf serum in PBS by the amperometric microfluidic array after incubation of Ab_2-_MB-HRP-analytes in detection chamber, then injecting mixture of H_2_O_2_ and HQ: (**A**) duplicate reproducible peaks in simultaneous measurements of a mixture of 10 fg mL^−1^ IL-6, 15 fg mL^−1^ IL-8, 25 fg mL^−1^ VEGF, and 60 fg mL^−1^ VEGF-C, (**B**) VEGF peaks in mixtures of all biomarker proteins. (**C**–**F**) Immunoarray calibrations of background corrected peak currents for IL-6 (**C**), IL-8 (**D**), VEGF (**E**), VEGF-C (**F**). Error bars are standard deviations for n = 6. Reprinted from [26]. Copyright 2012 American Chemical Society.

**Figure 4:**
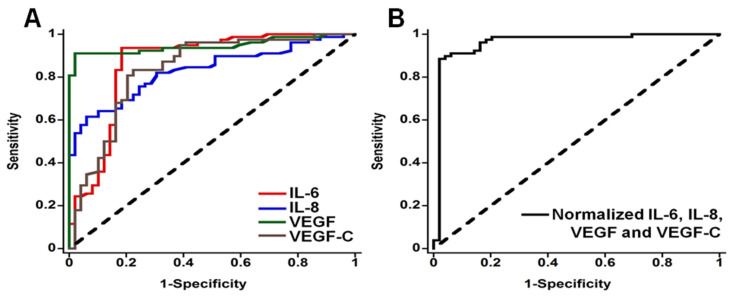
Receiver operating characteristic (ROC) curves for serum assays for (**A**) IL-6, AUC 0.86 (red line), IL-8 with AUC 0.83 (blue line), VEGF with AUC 0.95 (green line), VEGF-C with AUC 0.83 (brown line), and (**B**) mean normalized value for all four proteins, with AUC 0.96. Reprinted from [26]. Copyright 2012 American Chemical Society.

In 2012, we reported an amperometric microfluidic immunoarray for four proteins and evaluated it for oral cancer detection by analysis of patient serum [26]. Proteins in samples or standards are captured on 1 μm magnetic beads with 400,000 horseradish peroxidase (HRP) labels and 120,000 detection antibodies (Ab_1_). Beads are separated magnetically and introduced into the microfluidic array with eight nanostructured sensors with capture antibodies (Ab_1_) attached (Figure 2), and when the detection chamber is filled with this sample a 20 min stop-incubation period is initiated. For detection, a mixture of H_2_O_2_ and hydroquinone (HQ) mediator is injected into the sensor array to generate amperometric peaks for the reduction of HRP oxidized by H_2_O_2_ (Figure 3). H_2_O_2_ activates the Iron Heme of HRP to a Fe^IV^ = O form that is easily reduced with the aid of HQ as an electron mediator. This strategy provides the peaks seen in Figure 3 as the injected H_2_O_2_/HQ solution passes across each sensor in the array. This method provided very low LODs of 5–50 fg mL^−1^ for IL-6, IL-8, VEGF and VEGF-C proteins in dilute calf serum in 50 min. Good correlations with individual ELISA assays was found for the proteins overexpressed into growth media of oral cancer cells. The measured levels of the four proteins were normalized and averaged for 78 oral cancer patient serum samples and 49 cancer-free controls. It is essential to consider the statistical power of these clinical investigations, i.e., the probability that the analysis will find an effect, e.g., here, the measured protein level or combined protein levels that are upregulated in the presence of a particular cancer type. Power analysis can estimate the minimum number of samples required for a given study. Often, biomarker validation information obtained using traditional centralized assays, coupled with sensor performance in surrogate samples, can be used to predict the number of patients and controls required in the clinical phase. For our 4-plex assay, receiver-operator characteristic (ROC) analysis showed that normalized levels gave clinical sensitivity 89% (true positives) and specificity 98% (2% false positives) for oral cancer detection (Figure 4) [26].

Receiver operating characteristic (ROC) plots [27] are statistical analyses that assess diagnostic performance and can be done using MedCalc or R software. We used assay results and dependent variables of 0 for no cancer and 1 for cancer. Medcalc software identifies a decision threshold of the assay level above which cancer is predicted. Diagnostic value is judged by area under the curve (AUC), where AUC of 1.0 represents perfect prediction. Figure 4A shows curves for each of the four individual proteins in our panel in the patient samples. Figure 4B shows the ROC plot for the normalized average parameter, with AUC of 0.96, sensitivity 89% and specificity 98%. Results also showed that the panel can identify early oral cancers [26].

## 4. Predicting the Need for Prostate Cancer Biopsy

Aggressive prostate cancers having standard pathology Gleason score ≥8 are fast-growing, have 5-year survival rate 28%, and need timely treatment [28]. However, the majority of prostate cancers grow slowly, and do not metastasize (e.g., Gleason ≤ 6). These indolent cancers have 5-year survival rate of 99% [28]. Current clinical screening does not easily identify patients with aggressive prostate cancers [29]. The PSA test and digital rectal exam (DRE) are used [28,29] with blood PSA >4 ng mL^−1^ suggesting the possibility of cancer. DRE is a manual test for prostate abnormalities that depends on experience and skill of the examiner [30]. If these screens detect abnormalities, a needle biopsy may be recommended to decide if surgery is needed. However, PSA has low specificity forprostate cancer, and can also be elevated due to prostate inflammation and benign prostatic hyperplasia. It is estimated that 75% of biopsies based on high PSA levels are unnecessary [31]. In addition, modern prostate biopsies use multiple needles, are painful, and can miss small tumors [32]. More accurate diagnostic tests are clearly needed to help make decisions about the need for a biopsy [17,33].

We evaluated an 8-biomarker panel of prostate cancer biomarker proteins including some specific for aggressive cancers [34]. These are PSA, vascular endothelial growth factor-D (VEGF-D) [35], gene fusion proteins ETS related gene (ERG) [36], Golgi membrane protein 1 (GOLM-1) [37], pigment epithelial derived factor (PEDF) [33], insulin-like growth factor-1 (IGF-1) [38], insulin-like growth factor binding protein 3 (IGFPB-3) [35,39], and serum monocyte differentiation antigen CD-14 (CD-14) [40]. The assay used the same microfluidic device as in Figure 2 but was equipped with two 8-biomarker detection channels and a flow direction switch after the injector that enables two assays on the same sample in rapid succession to detect the eight biomarker proteins in duplicate. A new detection strategy without magnetic beads was used. Samples were premixed with a streptavidin-HRP polymer and biotinylated-Ab_2_′s for all the proteins, which formed analyte protein–HPR–Ab_2_ complexes. These solutions were injected into the microfluidic system and peaks similar to those in Figure 3A,B are obtained from a similar amperometric measurement principle as fr the oral cancer biomarkers. However, the HRP polymer label with 400 HRP units per molecule gave significantly better sensitivity and better LODs below the fg mL^−1^ levels for the prostate cancer biomarkers. Serum samples were diluted 100-fold before the assay. The remainder of the assay was identical as in the above oral cancer study.

Serum samples from 130 prostate cancer patients were analyzed (Table 2). Calibrations for proteins had dynamic ranges of 5–6-fold log decades, and LODs were from 0.3 to 3.1 fg mL^−1^_._ To confirm accuracy standard spike-recovery in pooled human serum was done and recoveries were 83–128%

**Figure 5:**
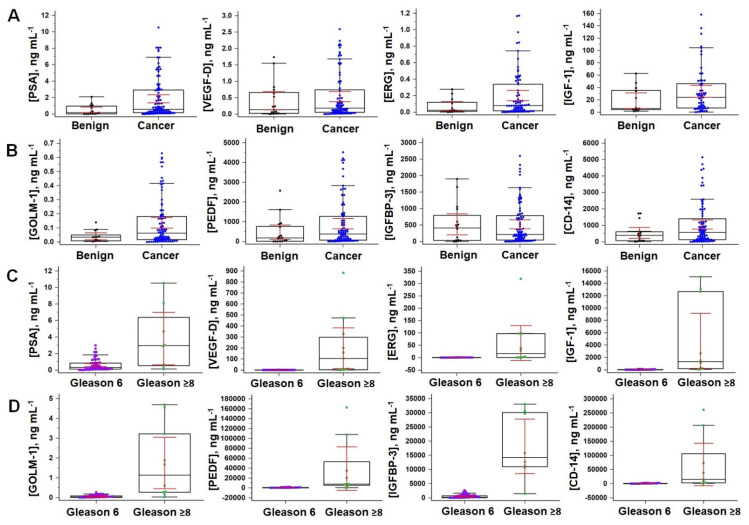
Box plots for patient samples. Protein biomarker levels in male patient serum (excluding outliers): (**A**,**B**) benign vs. cancer (high end outliers removed). (**C**,**D**) Indolent vs. aggressive (indolent—Gleason score of 6, aggressive—Gleason score >8). Error bars at 95% confidence. Reproduced from [34], copyright America Chemical society, 2021.

Assay data in box and whisker plots (Figure 5) show differences between samples with Gleason 6 and ≥8, but not benign and Gleason score > 6 (cancer). While these results are promising, due to low numbers of high Gleason samples, a much larger study is needed to validate identifying aggressive and indolent cancers. We thus focused on distinguishing if the protein panel could predict which patients required a biopsy. Logistic regression analysis of patient sample data was used to build a predictive model considering linear and logarithm of biomarker levels, but only one of these per protein biomarker. The clinical question “*Does the patient need a biopsy?*” was formulated as distinguishing between benign prostate disease and cancers with Gleason scores > 6 (Table 1).

In logistic regression, a dependent variable is given the value 1 (TRUE, i.e., do the biopsy) or 0 (FALSE, do not do the biopsy). Proteins were grouped into models using stepwise selection, and individuals with low significance were removed [41]. Models were PSA alone (model 1), PSA + log(VEGFD) + log(ERG) + log(IGF1) + PEDF +log(CD14) (model 2), and log(VEGFD) + log(IGF1) + PEDF + log(CD14) (model 3) [34].

**Figure 6:**
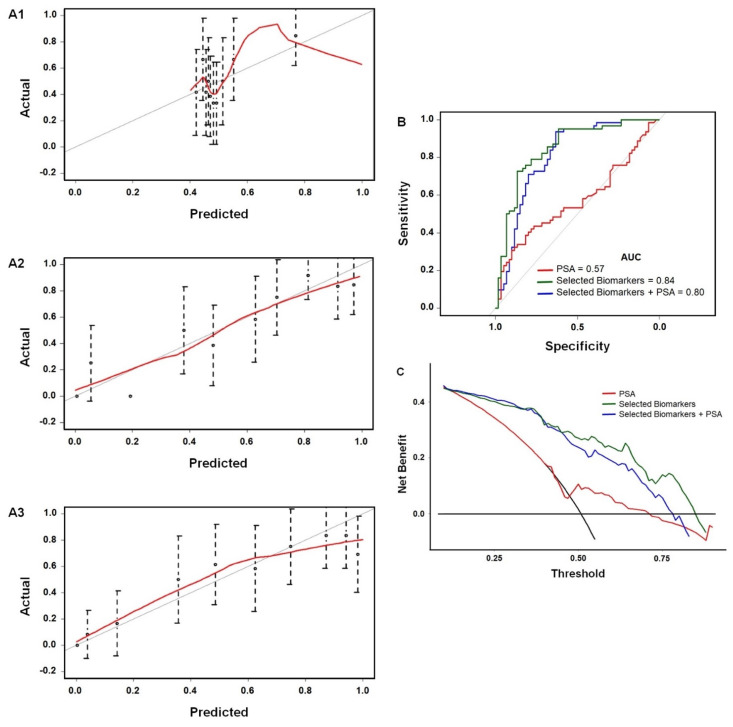
Calibration plots, ROC, and decision curves for predicting need for biopsy. Calibration plots illustrate predicted probabilities on the x-axis and the actual outcome on the y-axis (**A1**) PSA alone, (**A2**) selected biomarkers + PSA, model 2, and (**A3**) selected biomarkers, model 3. (**B**) ROC plot and AUC values of the biomarkers at each risk threshold, for the best model PSA + selected biomarkers. sensitivity and specificity are both 79%; (**C**) decision curves showing the net benefit of treating all patients or treating none vs. threshold probability. Reproduced from [34], copyright American Chemical society, 2021.

A Loess smoothing fit [42] (red line) along the 45° line shows the model is well-calibrated, as found for all the models (Figure 6A1–A3). ROC plots (Figure 6B) show that the biomarker models 2 and 3 are all significantly better at predicting necessity of a biopsy than PSA alone. Decision-curves (Figure 6C) [34] reveal net benefit vs. threshold probability of a patient opting for a biopsy. Again, models 2 and 3 are much more informative predictors than PSA alone as they show a higher net benefit.

## 5. Detecting a Cell-Bound Metastatic Biomarker

In this section, we describe a microfluidic device coupled to a cell disruptor designed to measure biomarker proteins within cancer cells [43]. Our focus was on a system that could detect cancer metastasis since 90% of cancer deaths result from metastasis of original tumors [44]. The biomarker proteins were the cell-bound protein desmoglein 3 (DSG3), a metastatic biomarker for oral cancer [45] and VEGF-A, an oral cancer biomarker expressing outside the cancer cells (VEGF = vascular endothelial growth factor).

Here, we turned to 3D printing to design and fabricate a single-unit microfluidic immunoarray. Low cost desktop 3D printers (USD 1000–4000) offer revolutionary new options for rapidly designing, optimizing and fabricating high performance-low cost microfluidic array devices [46,47]. 3D printing is an excellent tool for designing and fabricating microfluidic diagnostic devices [48,49,50]. It provides prototypes rapidly and can facilitate design-to-fabrication times of several hours. Objects are designed by computer-aided design (CAD) to generate 3D-print instructions that are uploaded to printer memory. Fabrication proceeds layer-by-layer by laser processing of liquid photocurable resin precursors in the stereolithographic (SLA) printers used here. Optimization is achieved at a small fraction of the time and cost of lithography, and commercial devices can be mass-produced by scaled up facilities. Essentially, the optimized prototype becomes the final usable product. Low 3D-print development costs far outweigh the need for high feature resolution, which is usually not required. Table 3 lists diagnostic devices that have been developed by 3D printing.

**Figure 7:**
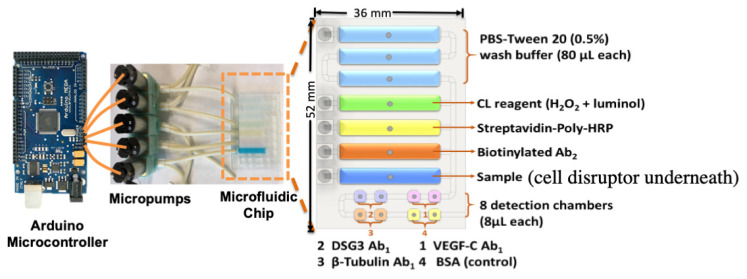
Microfluidic immunoarray for cell disruption and protein detection. The design features a microfluidic chip with five inlets connected to peristaltic micropumps, sample and rectangular prism reagent chambers with capacity of 80 ± 5 μL, and eight cylindrical detection chambers with 8 ± 1 μL capacity each. The microfluidic chip houses sample and reagents and delivers them sequentially to the detection compartment. The assay protocol utilizes poly-HRP and ultra-bright femto-luminol to produce chemiluminescence (CL) that is captured in a dark box using a CCD camera. The microfluidic chip is mounted on the housing device support equipped with a sonic cell disruptor that achieves cell lysis. Programmable micropumps are connected to microfluidic chip sample and reagent chambers and the assay is automated by using an Arduino microcontroller that control pump on/off cycles. Adapted from [43], with permission. Copyright Elsevier, 2021.

The problem we needed to solve was how to incorporate cell disruption into a microfluidic immunoassay device. After several unsuccessful attempts, we designed, fabricated and tested the device in Figure 7. We used chemiluminescence (CL) for detection since it produces bright light, is amenable to camera, or even iphone camera, detection, and does not need other external instrumentation. Thus, the device should be compatible with POC use.

We designed a disposable microfluidic chip with five separate chambers for reagents and samples, each with its own programmable peristaltic micropump (Takasago Fluidic Systems) for delivery to chamber containing a connected series of cylindrical detection wells. Individual pumps were used to avoid clogging by cell debris if only one pump was used and move all the solutions through a linear train of chambers. A Formlabs Form 2 3D SLA printer was used to print the device from polyacrylate clear resin for USD ~0.60/unit. Sample and solutions of biotinylated antibodies, poly-HRP, ultrabright CL reagent femto-luminol and buffer (PBS-Tween20) (Figure 7) were delivered in the correct sequence to the detection compartment with eight microcylinders (8 μL vol.) filled with chitosan hydrogel with Ab_1_ antibodies attached. These were outfitted with the correct Ab_1_′s to measure the proteins DSG3, VEGF-A, and β-tubulin, and a negative control (BSA). The micropumps are activated to deliver sample and reagents to detection microcylinders by an Arduino^®^ microcontroller preprogrammed with the optimized pump on-off and incubation stopped flow timing. Individual micropumps are housed in a 3D-printed support that includes a SonicSoak cell lysis probe attached directly under the sample chamber. Sonication for 2s for cell samples diluted in RIPA lysis buffer gave the optimum cell disruption. The detection mechanism is similar to that in the prostate cancer biomarker assay above, but here poly-HRP-Ab_2_-protein analyte aggregates are formed, and they binds to partner Ab_1_’s in their specific detection cylinders. The HRPs are then activated by H_2_O_2_ to form HRP-Fe^IV^=O which oxidized femto-luminol when present to produce a product that spontaneously emits visible chemiluminescence (CL). The light is measured in a dark box with a CCD camera [43].

For calibrations, pure proteins were dissolved in RIPA lysis buffer, which is 50 mM Tris-HCl, 150 mM NaCl, pH 8.0, 1.0% Igepal CA-630, 0.5% Na deoxycholate, 0.1% SDS, 2% Halt Protease-Phosphatase Inhibitor Single-Use Cocktail in 5-fold diluted pooled human serum.

**Figure 8:**
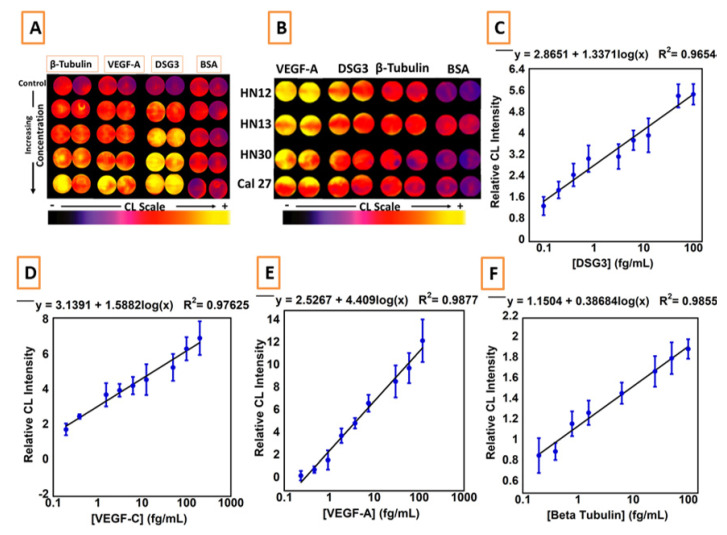
Recolorized CL CCD camera for 15 s integration for protein analytes in (**A**) standards and (**B**) cancer cell cultures. (**D**–**F**) Calibrations of the microfluidic immunoarray for (**C**) DSG3, (**D**) VEGF-C, (**E**) VEGF-A, and (**F**) β-Tub (n = 8). Adapted from [43], with permission. Copyright Elsevier, 2021.

Figure 8 shows biomarker protein calibration data obtained with this device. CL CCD images of standard proteins detected in oral cancer cell cultures are shown (Figure 8A,B). LODs were 0.10 fg/mL for DSG3, 0.20 fg/mL for VEGF-A. β-Tubulin has relatively constant levels in oral cancer cells, and is used as a loading control in Western blots [45]. We included it in the assay to be used as a cell number metric for highly diluted samples in results discussed below.

Here, we discuss detection of the biomarker proteins in single cells [43]. Oral cancer cell cultures having a known number of cells per unit volume were diluted until only an estimated one cell remained in 10 μL. Due to statistical variations, these highly diluted samples may have zero cells, one single cell, or more than one cell.

We measured β-Tubulin content per cell for undiluted samples for counting the numbers of cells. The number of cells in highly diluted samples were then found from their β-Tub level divided by β-Tub conc. per cell. First, the cells in the cultures were filtered, washed and resuspended in osmotically balanced PBS and determined by the cell disruptor-immunoarray. These measurements gave protein levels in the cells only. Significant amounts of VEGF-A and DSG3 appeared in the washed cells. VEGF-A, DSG3, and β-Tub were then measured by the cell disruptor-immunoarray in washed cells diluted to achieve approximately one cell/10 µL.

**Figure 9:**
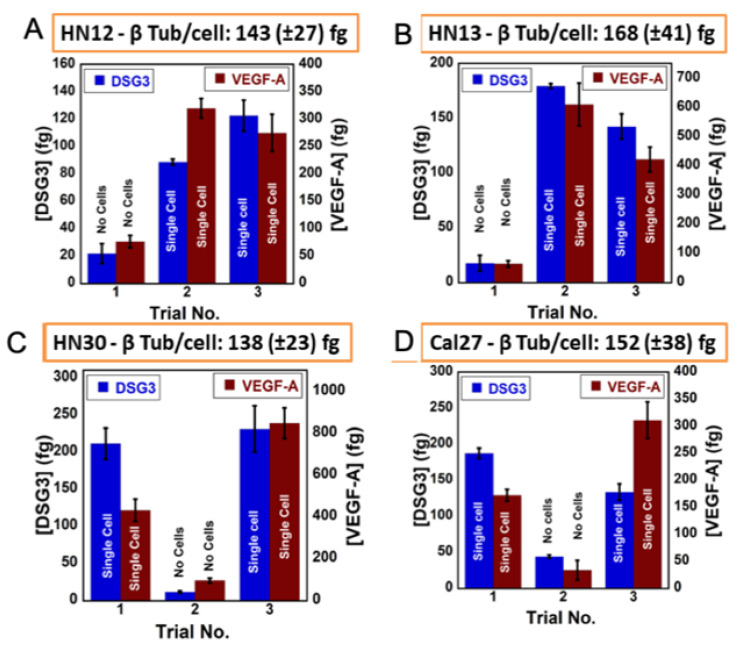
Protein concentrations found using online cell lysis of single cell or cell-free samples filtered and washed to remove cell culture media for cancer cell cultures (**A**) HN12, (**B**) HN13, (**C**) HN30, and (**D**) CAL27 (n = 6). Number of cells was estimated using the β-Tub concentration per single cell. Zero cell or single cell samples are marked on the graphs for DSG3 and VEGF-A. Adapted from [43], reprinted with permission. Copyright Elsevier, 2021.

The amounts of DSG3 and VEGF-A were estimated in single cell samples as confirmed by the β-Tub cell marker (Figure 9).

This 3D-printed cell disruptor-immunoarray is the first sub-fg detection assay with on-chip lysis for protein measurements in single cells. Since it is automated, it can be operated by relatively untrained personnel. We envision future applications to lymph node tissue for cancer metastasis diagnostics.

## 6. Label Free Electrochemical Detection of Cancer Cells

The ability to detect rare cells within a patient’s blood, e.g., circulating tumor cells, CTCs, in cancer patients or low numbers of pathogens in the early stages of sepsis, using point-of-care devices would represent a significant advance in contemporary clinical practice [62,63,64,65,66]. However, the analytical challenge is significant in the sensitivity and limit of detection, LOD, required, e.g., 5–10 CTCs per mL, is a concentration of 10^−19^ M! Moreover, extreme selectivity is required due to the high concentrations of red and white blood cells and platelets present in whole blood. The difference in concentration between the target and background cells is at least one million-fold, i.e., the association constant for the bioreceptor that binds the target cell needs to be 10–100 million-fold higher than for other cell types in the sample. These extreme demands of sensitivity and selectivity mean that complex analytical strategies are often required, but portable, point-of-care devices capable of rapidly detecting and identifying cells are emerging steadily [67,68,69,70,71].

Electrochemical detection methods that use a label, e.g., a redox active molecule such as ferrocene, or an electrocatalytic nanoparticle such as platinum, tend to be more sensitive. However, electrochemical impedance can be used to sensitively measure subtle changes in interfacial impedance when a target binds to an immobilized bioreceptor [72]. In particular, the cell resistance (intersection of the right-hand side of the Nyquist semicircle with the x-axis) and the interfacial capacitance can depend on surface coverage of the capture agent and the target. For example, binding of the target cells to the antibody modified capture surface may impede ion transport, thus increasing the overall resistance of the electrochemical cell. Alternatively, cell binding may displace ions and solvent from the electrode surface decreasing the interfacial capacitance, but sometimes the opposite effect is observed because the biological cells are charged and bring additional ions upon binding.

**Figure 10:**
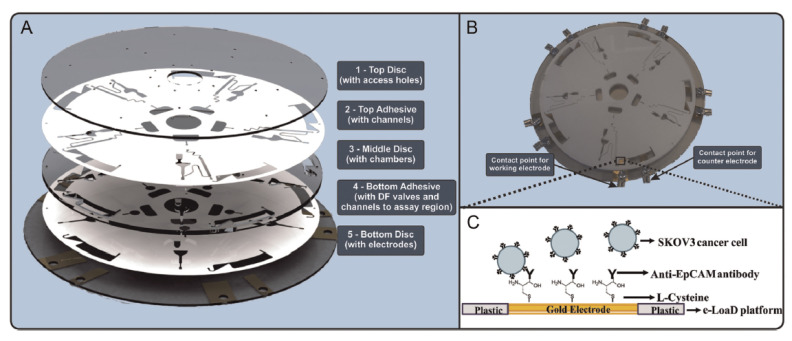
(**A**) Rendered 3D image of the five-layer microfluidic disc platform comprising three 1.5-mm thick PMMA discs and two 90-μm thick pressure sensitive adhesive films. The gold electrodes were deposited on the bottom (layer 5) of the disc. (**B**) Fully assembled disc showing contact points for the working and counter electrodes. (**C**) Schematic of electrochemical cancer cell capture assay on polymeric eLoaD platform. Reproduced with permission from [76].

An advantage of label free detection is that genetic or protein expression profiling can be performed on the captured cells to obtain powerful prognostic information [73]. Significant accomplishments have been made in the detection of cancer cells [74,75,76,77] where selective detection can be accomplished using capture antibodies, peptides or aptamers and an optimized detection frequency are used so as to maximize the sensitivity of the impedance change. As illustrated in Figure 10, combining the benefits of electrochemical impedance spectroscopy (EIS) and Lab-on-a-Disk (LoaD) platforms, has allowed us to detect ovarian cancer cells without the need for labeling, e.g., with a fluorescent dye, in a fully integrated platform [76,77].

On this platform, the capture of cancer cells causes the electrode capacitance to decrease as well as causing a minor increase in the resistance. Cell binding is expected to trigger displacement of ions and solvent at the interface thus causing the capacitance to decrease. However, it is important to note that the state of charge of the interface after cell binding will depend on the specific properties of the cells being bound, e.g., the coverage of protein receptors, their isoelectric point and the pH/ionic strength of the measurement buffer. Therefore, these label-free electrochemical strategies may need the measurement and sample conditions to be more rigidly controlled than label-based approaches. However, with optimized conditions a reliable impedance change can be detected when only 2% of the electrode surface is covered with cancer cells. An important but under reported aspect of POC devices is the overall capture efficiency that is influenced by the microfluidic transport of the cells to the electrode surface, the cell capture rate and the overall bioreceptor-cell binding energy. The capture efficiency will influence the LOD since cells must be immobilized in order to be detected. It will also affect the dynamic range since a very high capture efficiency can cause the surface to be fully covered with captured cells even though the concentration in suspension is low. For the eLoaD device shown in Figure 10 the dynamic range is from 2.6 × 10^6^ to 1.6 × 10^10^ cells/mL.

## 7. Sample to Answer Microfluidic Device for Neurological Disease

### 7.1. Temporal Lobe Epilepsy

The clinical diagnosis of temporal lobe epilepsy (TLE) relies on electroencephalogram (EEG) or video-EEG as well as patient history of seizures because validated, blood-based molecular biomarkers are lacking. MicroRNAs, miR, have a high degree of cell-specific expression, demonstrated mechanistic links to brain excitability, and are present in blood, cerebrospinal fluid and even perspiration. Using a large cohort of patients that included individuals suffering from psychogenic non-epileptic seizures (PNES), miR-27a-3p, miR-328-3p and miR-654-3p have demonstrated significant potential as biomarkers of TLE [78,79].

A point-of-care device, TORNADO, that is based on a spinning disk (similar to a CD or DVD) has been developed for the rapid detection of these microRNAs in plasma [78]. This work used an electrocatalytic nanoparticle as the label. This strategy maximizes the signal-to-noise ratio since the underlying electrode is chosen to have a very poor performance for the electrocatalytic reduction of the substrate, e.g., hydrogen peroxide, while the nanoparticle labels can reduce H_2_O_2_ at a diffusion controlled rate. This sensing principle brings two advantages. First, it is relatively easy to create an electrode surface that is not electrocatalytic for the reduction of hydrogen peroxide which minimizes the background or “noise”. Second, even a single highly catalytic particle associated with a single target molecule binding event can generate a theoretically measurable current, thus maximizing the signal generated. In this way, electrocatalytic nanoparticle can maximize the S/N ratio that is the key requirement for ultrasensitive detection of disease biomarkers. The gold working electrode was first functionalized with thiolated capture miRNA that is complementary to part (approximately 50%) of the target. The multichamber disc is then loaded with the plasma sample (100 μL) the complementary probe functionalized platinum nanoparticles, as well as buffers for the wash and measurement steps. Dissolvable valves within the disc are used for sequential, event-triggered release of the reagents required for each step, i.e., the arrival of liquid at one location drives the release of a second liquid at another location [80]. Depending on the stage of the test, e.g., sample introduction, wash step, measurement buffer introduction, the disc spins at 1–35 Hz to drive the appropriate liquid into the measurement chamber. The plasma sample was incubated for 30 min in contact with the capture strand modified gold electrode to allow hybridization to occur before being washed with buffer to remove physisorbed material. Then, a 1 μM suspension of the 50 nm platinum nanoparticles functionalized with probe miRNA; that can bind to the “overhang” of the target was added and allowed to hybridize for 30 min. Finally, the measurement chamber was filled with a hydrogen peroxide solution that acted as both a wash step and the measurement buffer. The number of nanoparticles on the surface of the electrode depends on the concentration of target miRNA in solution and the surface concentration of the nanoparticles, Γ_NP_, controls the magnitude of the electrocatalytic current generated. The theoretical current associated with a single nanoparticle under radial diffusion conditions (nanoparticles are isolated from one another, i.e., target concentration is low) is approximately 2 nA where the peroxide concentration is 100 μM. Thus, single binding events cannot be discriminated against the capacitive and parasitic faradaic currents at the underlying electrode. However, it is possible to detect the electrocatalytic current associated with the nucleic acid hybridization mediated binding of a few thousand particles, i.e., the binding of a few thousand copies of the microRNA target.

It is important to note the nature of the mass transport by diffusion to the electrode surface can change depending on the concentration of miRNA in the sample and hence the coverage of the electrocatalytic nanoparticles. For example, when the concentration of miRNA in the sample is low, Γ_NP_ is low and the well-separated nanoparticles act like a micro- or nanoelectrode array with non-overlapping, radial diffusion fields. However, depending on the timescale of the current measurement, at intermediate miRNA concentrations, these depletion zones may begin to overlap giving mixed radial and linear diffusion control. Finally, when the gold electrode surface is completely covered by platinum nanoparticles, the depletion zones will overlap at all timescales and linear diffusion will dominate. Unexpectedly, given that radial diffusion is much more efficient than linear diffusion, these differences in mass transport regime can cause the current observed to be lower for the samples that contain more target miRNA and the data must be interpreted carefully. One useful strategy is to change the area of the gold electrode so that the surface coverage of the platinum nanoparticles is controlled and the diffusion regime does not change even though the concentration of the target changes significantly.

Quantitation of the microRNAs within the exosome-enriched fraction provided a high diagnostic accuracy. However, many microRNAs will be protein bound and the miR-328-3p that is bound to argonaute was found to increase selectively in patients after seizures. In situ hybridization demonstrated that miR-27a-3p and miR-328-3p are localized within neurons in the human brain and bioinformatics predicted targets linked to growth factor signaling and apoptosis. It is important to emphasize that sample-to-answer, point of care devices can be powerful for diagnosis, prognosis and understanding the mechanistic links to underlying pathomechanisms, but only if validated biomarkers are used.

### 7.2. Mild Cognitive Impairment and Alzheimer’s Disease

Another example where early diagnosis remains challenging due to a lack of reliable biomarkers is Alzheimer’s disease (AD). However, blood-based microRNAs also have significant potential as diagnostic and even prognostic biomarkers for AD. For example, Let-7b and miR-206 have been validated to be at higher levels in patients suffering mild cognitive impairment (MCI) or AD. Significantly, Let-7b levels in plasma may identify patients with MCI, while monitoring the plasma levels of miR-206 may predict cognitive decline and progression towards dementia at the mild impairment stage.

**Figure 11:**
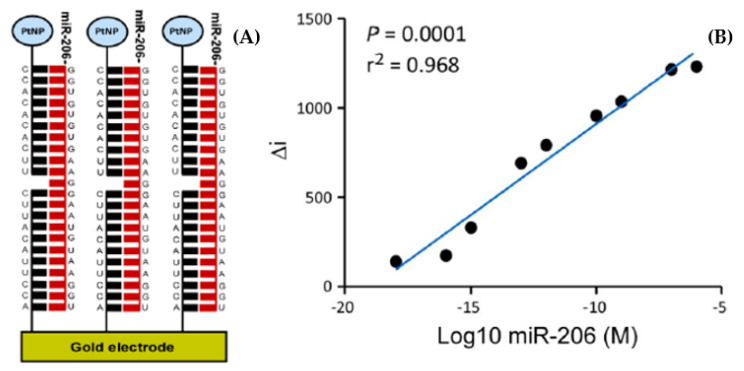
(**A**) Schematic representation of the nucleic acid sandwich assay using electrocatalytic nanoparticles as labels on the probe strand. (**B**) Calibration curve for difference in current (Δi) before and after injection of hydrogen peroxide against known concentration of miR-206 oligonucleotides (linear between 100 nM and 100 aM). Reproduced with permission from [79].

As illustrated in Figure 11, we have used our sample-to-answer centrifugal disk platform to detect the miR-206 target [79]. These nanoparticles electrocatalytically decompose hydrogen peroxide that is added after the target has hybridized with the capture strands immobilized on the electrode surface. Figure 11B shows that the difference in current observed before and after the addition of hydrogen peroxide depends linearly on the target concentration from 100 aM to 100 nM.

The wide linear dynamic range is controlled by the electrode area and how closely the nanoparticles can pack, while the limit of detection is dictated by the current generated by an individual functionalized nanoparticle relative to the background current of the noncatalytic electrode itself.

This work used qPCR to validate the miR-206 biomarker itself and the performance of the sample-to-answer disk. It demonstrated that upregulation of miR-206 is strongly correlated with cognitive decline and memory deficits and that increases in miR-206 precede the onset of dementia potentially allowing high risk individuals to receive therapy earlier. Microfluidic devices of this type, that reduce the level of operator expertise needed through reagent preloading and event triggered release could provide a practical cost-effective tool for the stratification of patients with MCI according to risk of developing AD. However, it is important to note that for mass manufacture smart strategies for calibration and appropriate positive and negative controls need to be included on the device.

## 8. Biomarkers of Cardiovascular Disease

As discussed above, cancer cells can be detected using label free electrochemical impedance spectroscopy (EIS). The capture of a small number of cells generates a measurable change in the interfacial capacitance that can then be detected using AC impedance. Where the target is molecular, e.g., a biomarker of acute myocardial infarction, AMI, cardiac troponin, cTn, the sensitivity can be significantly enhanced by using a redox active label. cTn is an important biomarker of acute coronary syndrome. It exists as a trimer comprising three single-chain polypeptides: troponin T, cTnT, that binds the other troponin components to tropomyosin, troponin I, cTnI, inhibits ATP activity when bound to actin, and troponin C, cTnC) contains binding sites for calcium. Typically, following AMI, the troponin complex breaks apart releasing the individual protein components into the bloodstream. cTnI is more specific for cardiac related events [81] and reaches a maximum concentration of the order of 50 ng/mL during the 24 h following AMI [82]. Thus, there is an unmet clinical need for sensors or microfluidic devices that can sensitively and selectively detect cTnI within a short (tens of minutes) period at the point of need, e.g., by emergency response teams. EIS detection often uses a redox probe in solution whose heterogeneous electron-transfer rate constant is affected by the binding of the antigen to the immobilized antibody, i.e., there is a change in the charge transfer resistance. However, adding a solution phase probe may change the biological system, e.g., cause conformational changes in the antibody or antigen or bind to other components in the sample, changing the sensitivity of the assay. Labeling the secondary antibody minimizes the total quantity of the label needed and allows changes induced by labeling to be more completely understood ex situ.

**Figure 12:**
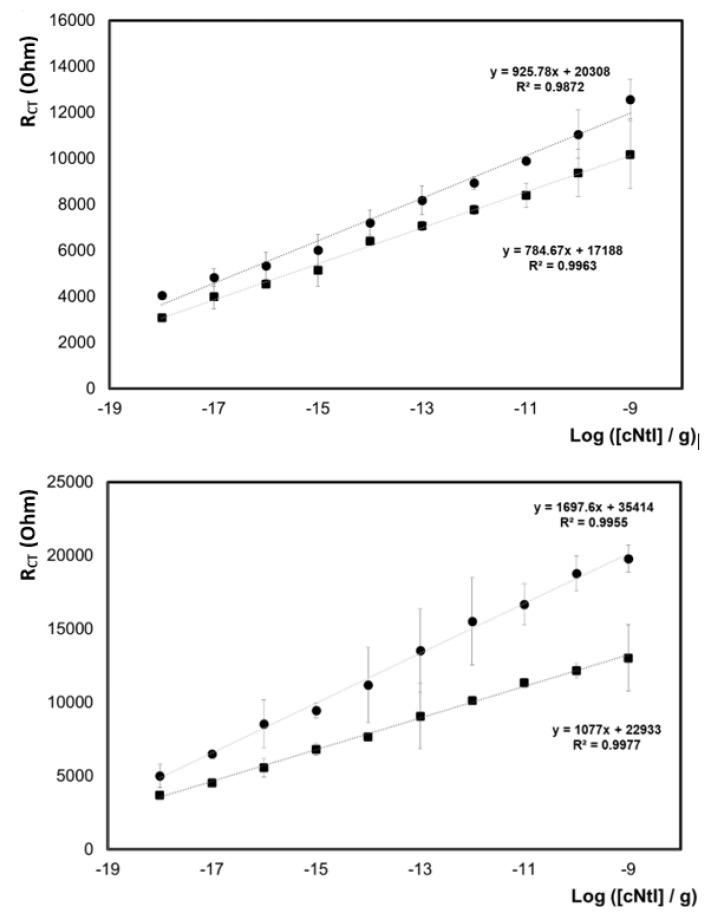
(**A**) Dependence of interfacial resistance (n = 3) on cTnI concentration where the primary antibodies are mAb20B3 (●) and mAb228 (■), following exposure to increasing cTnI target (1 ag/mL to 1 ng/mL) and after immobilization of the Ir (III)-labeled commercial secondary antibody mAb19C7 (**B**). In all cases, the supporting electrolyte is 1 mM DPBS and EIS was recorded between 0.01 and 100,000 Hz using an alternating current (ac) amplitude of 25 mV. Reproduced with permission from [83].

Figure 12 shows an assay for the sensitive and selective detection of cTnI using a custom capture antibody and a commercial secondary antibody labeled with a carboxy-functionalized cyclometallated iridium complex that acts as a label in faradaic electrochemical impedance [83]. It is essential to consider the physical dimensions over which the target binding occurs so that the length scale over which the interfacial electric field decays (double layer thickness) and the binding region are comparable. This maximizes the impedance change upon target binding while minimizing the impact of changes in the solution phase composition between individual real-world samples. For the cTnI assay a dilute electrolyte, 0.001 M DPBS, was used. EIS can reveal changes in both the interfacial capacitance, e.g., due to a change in the interfacial ion distribution or effective dielectric constant, or the charge transfer resistance.

**Figure 13:**
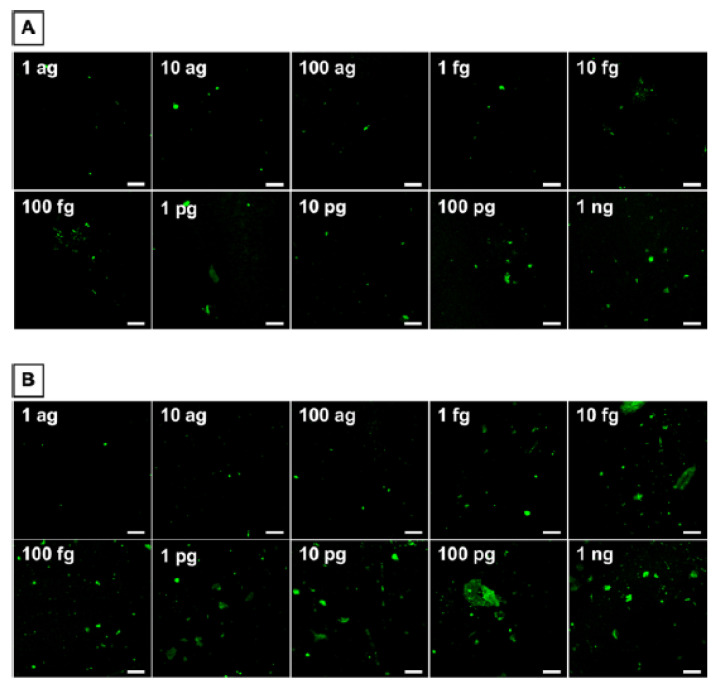
Confocal images of an electrode modified with 16-mercaptohexadecanoic acid and the in-house-generated mAb20B3 (**A**) and a commercially available Hytest mAb228 primary antibody (**B**), following exposure to the cTnI target (1 ag/mL to 1 ng/mL) and the Ir(III)-labeled commercial secondary antibody (mAb19C7). Luminescence images were recorded live on a Zeiss LSM510 Meta confocal microscope using a 40× oil immersion objective lens (NA 1.4) and a 488 nm argon ion laser applied for iridium-labeled antibody imaging. Scale bar 20 μm. Reproduced with permission from [83].

Figure 12 shows that the resistance depends linearly on log[cTnI]. Linear semi-log concentration vs. sensor response are often reported in the literature but are not always consistent with the expected direct correlation between response and analyte concentration predicted for amperometric and spectroscopic techniques. Here, it suggests that the impedance response is influenced by more than one process, e.g., the successful binding of Iridium labeled secondary antibody as well as the cTnI concentration. Significantly, the iridium complex is luminescent which, as shown in Figure 13, allows the binding of the Ir-labeled secondary antibody to be visualized.

Experiments of this type that can independently quantify each step in the overall sensing strategy are extremely important, e.g., determining the surface coverage of the capture antibody, as well as the dynamics (rate) and thermodynamics (association constant) of both target and secondary antibody binding. The fluorescence intensity is not uniform across the images which could arise from a non-uniform deposition of the capture antibody or perhaps some aggregation/preferential binding sites for cTnI. Effects of this type are likely to be significantly influenced by electrostatic interactions, i.e., it is important to consider the isoelectric point, pA, of the various components under the deposition and analysis conditions. For both the custom and commercial antibodies, the emission intensity increases with increasing troponin concentration. Significantly, the fluorescence intensity for 1 ng cTnI with the Ir−mAb19C7 antibody was 30% higher for mAb20B3 capture antibody compared to the Hytest mAb228 one. This enhanced emission at low concentrations is potentially useful for the early detection of cTnI and could be translated to an electrochemiluminescence based platform. Overall, the results show that the capture antibody mAb20B3 has an extremely high affinity for cTnI. However, it is always valuable to quantify the impact of surface immobilization on the association constant and to understand how local changes in the composition of the capture layer, e.g., state of charge, hydrophilicity, dielectric constant, etc., influence both K_a_ as well as the on and off binding kinetics.

Electrochemiluminescence (ECL), in which an electronically excited state is electrochemically created that then goes on to emit light, enables the sensitive and selective detection of biomolecules with exceptional signal-to-noise ratios because of the dark background. ECL is simpler to implement than optical excitation since an excitation source is not required and the response can be turned on and off by controlling the applied potential. The response is only generated close to the electrode surface which offers enhanced discrimination over solution phase interferences [84,85]. A key challenge is the design of the ECL luminophore that needs to be able to undergo highly exergonic electron transfer reactions leading to the creation of an electronically excited state that can then emit. Identifying materials, especially metal complexes, that can satisfy these thermodynamic requirements while simultaneously allowing the wavelength and voltage of the electrochemiluminescence to be tuned, e.g., to avoid absorbance and redox active components of whole blood, is challenging. Thus, for inorganic luminophores, polypyridine type complexes of metals such as ruthenium, osmium and iridium continue to be important. For example, the hydrophobicity, physical location of the excited state within the complex, luminescence lifetime, wavelength of maximum emission and both ground and excited state redox potentials can be optimized for particular applications by changing the identity of the peripheral ligands. These changes directly impact the overall performance of assays, e.g., longer excited state lifetimes may lead to a brighter ECL response making the assay more sensitive, while reducing the redox potentials may eliminate parasitic Faradaic reactions thus making the assay more selective, or maximizing the rate of heterogeneous electron transfer across the electrode/solution or electrode/film interface can lead to a greater number of light emitting events per unit time. However, it is important to note that the photoluminescence efficiency does not directly predict the ECL intensity because ECL generation involves multiple mass transport and electron transfer processes, any of which can be the rate determining step. Moreover, there is often no obvious relationship between the photoluminescence quantum yield and the ECL intensity. For similar reasons, the use of multiple emitters within a single label, e.g., a dendrimer or metallopolymer (nano)particle, typically leads to an ECL intensity that is somewhat less than the sum of the intensities of separate, discrete luminophores and the extent of electronic coupling between the ECL luminophores needs to be carefully controlled.

By acting as coreactants in the creation of the excited state, a rather diverse range of biologically relevant molecules, e.g., the nucleic acid guanine and oxidation products, and some amino acids, therapeutics and drugs can be directly detected [86,87,88]. However, coreactant ECL in which a molecule such as tri-propyl amine is oxidized to generate a highly reactive cation radical that then reacts with an electrogenerated metal complex, e.g., [Ru(2,2′-bipyridine)_3_]^3+^, to create the reduced metal complex but in an electronically excited state [89], remains important. This approach has been successfully commercialized and some platforms offer assays for more than 100 analytes. Many of these are antibody-based sandwich-type assays for protein biomarker detection and typically use a capture antibody immobilized on an electrode surface that selectively enriches the concentration of target antigen at the sensor surface. A secondary antibody, labeled with an ECL generating luminophore, then binds to the captured antigen and the intensity of the light generated is proportional to the analyte concentration.

Recombinant antibodies, e.g., scFv fragments that contain peptide linkers between the VL and VH chains, are emerging as highly sensitive capture agents that can produced more rapidly than conventional antibodies in animals. These systems also have other advantages, e.g., broader linear dynamic range and lower nonspecific binding due to their smaller physical dimensions, as well as functional groups can be expressed that facilitate immobilization perhaps even in an optimum orientation for target binding. It is perhaps important to note that the smaller space occupied on the surface is unlikely to improve sensitivity since at low analyte concentration the availability of binding sites is unlikely to be the factor that dictates the intensity of the ECL generated. At high analyte concentrations where the luminophores may be at close proximity to one another, it is possible for electron transfer to occur from the electronically excited luminophore to an adjacent oxidized label in the ground state. This process will quench the emission causing the target concentration to be underestimated at high concentration and perhaps lead to nonlinear calibration curves.

**Figure 14:**
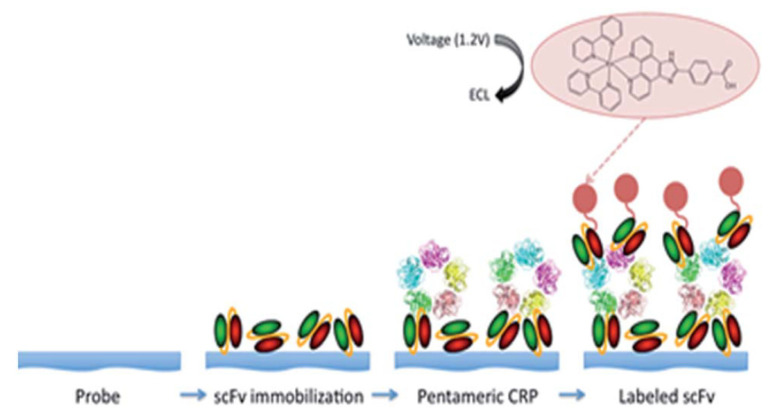
Schematic illustration of the fabrication of recombinant antibody-based biosensor for the detection of C-reactive protein. First, scFv fragments are immobilized on the electrode surface. Second, the pentameric CRP target binds. Third, scFv fragments that are labeled with a ruthenium polypyridine type metal complex are bound and used to generate ECL. Reproduced with permission from [90].

We have developed a high-performance electrochemiluminescent assay for the cardiac biomarker C-reactive protein (CRP) using recombinant antibodies [90,91]. CRP is an indicator of inflammation, but a higher level is associated with an increased risk of stroke and myocardial infarction. Figure 14 shows the steps involved in the assay. The purified recombinant scFv antibodies have a high affinity for monomeric C-reactive protein (mCRP, K_D_ 3.53 × 10^−10^ M) and can be effectively immobilized on platinum electrodes which are less susceptible to corrosion, e.g., due to chloride ions in PBS buffer or as an adventitious impurity from a leaking reference electrode!

**Figure 15:**
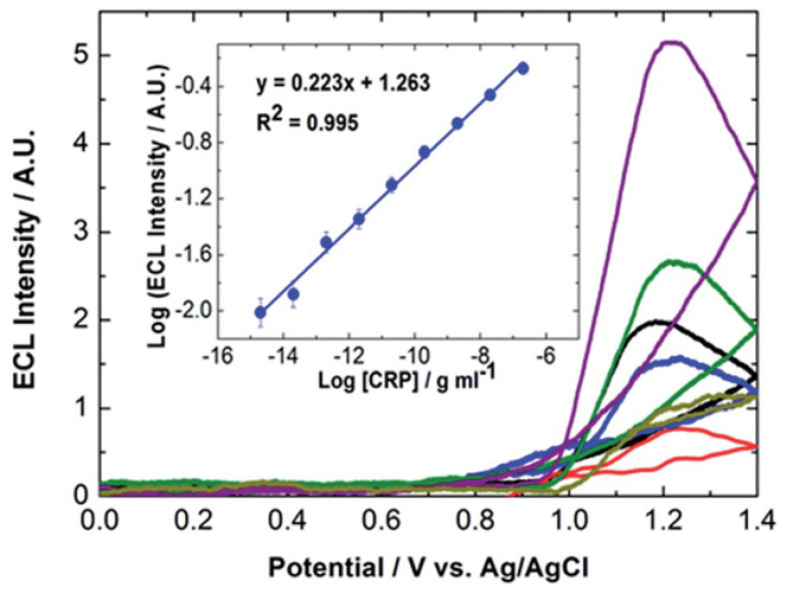
Dependence of the ECL emission intensity on the CRP concentration. From top to bottom at +1.2 V, the concentrations of CRP range from 600 ng mL^−1^ to 5 fg mL^−1^. Not all ECL responses are shown. The inset shows dependence of the logarithm of the maximum ECL intensity on log[CRP]. The error bars are comparable to, or smaller than, the size of the symbols. Reproduced with permission from [90].

Figure 15 shows that the ECL intensity of ruthenium complex bound to the secondary scFv fragment reaches a maximum intensity at approximately +1.2 V which is positive of the formal potential, E^o’^, of the Ru^2+/3+^ couple, +1.05 V. When using TPA as a coreactant, it is possible for TPA radicals to diffuse away from the electrode where they are generated and react directly with the ECL luminophore to generate the excited state. This process allows ECL to be generated at potentials that are less positive than E^o’^ (Ru^2+/3+^), e.g., +0.8 V. The absence of ECL at low potentials in Figure 15 indicates that this process is not important in generating light most likely because of the relative rates of mass and charge transfer in this system which is related to the size of the bioreceptor. Figure 15 shows that the ECL emission intensity depends linearly on the CRP concentration from approximately 5 fg mL^−1^ to 600 ng mL^−1^.

The upper limit of the dynamic range, 600 ng mL^−1^, is within the clinical range for low risk levels of CRP. More importantly for the broader development of ECL assays using recombinant antibodies, the limit of detection (LOD, average ECL emission of the blank + 3SD (blank)) was 0.3 fg mL^−1^ which is considerably lower than the pg mL^−1^ achieved by commercially available CRP testing kits or the ng mL^−1^ LODs typically achieved using electrochemical detection [92,93]. Overall, this work demonstrates that combining high-performance recombinant antibodies with ECL detection represents a powerful strategy for the selective detection of disease biomarkers at low concentration.

## 9. Conclusions and Future Outlook

Biosensors for clinical applications is a strongly developing field that uses the latest innovations in electroactive hybrid and nanomaterials for enhanced detection sensitivity to provide early insights into disease. Both labeled and unlabeled electrochemical detection strategies continue to have distinct advantages over optical detection including simpler, more robust instrumentation at lower cost, very high sensitivity, portability, speed of analysis and low power demand. Chemiluminescence and electrochemiluminescence are also very attractive for detection since they can be measured with a CCD or phone camera to provide high sensitivity. It is frustrating that biosensor approaches to minimally invasive medical diagnostics have been slow at finding their way into clinics and hospitals. Tests and devices need to be accessible to all potential users at low cost, which in a practical sense means that commercial suppliers need to become involved. There is a strong need for deeper and more extensive academic–business–clinical (ABC) collaboration to enable meaningful testing with real clinical samples and point-of-use clinical trials/validation. Given their central role in personalized, decentralized healthcare at lower overall cost, there is a tremendous need for methods to be statistically robust with low false positive and false negative rates. Direct comparisons with “*gold standard*” approaches are needed and may require prospective clinical trials. These ABC partnerships would also act to raise the rather low technology readiness level, TRL (predominantly TRL 2-3, proof of concept or prototype), that can typically be achieved in an academic laboratory in a time efficient way. Key issues, such as mass manufacturability at low cost, the ease of use for the individuals actually using the devices (ideally with minimal or zero training), reimbursement, adoption, reagent stability, cost of goods, clinical workflows, etc., are often not considered sufficiently early in the development process. While transitioning excellent diagnostic science to the clinic continues to pose many challenges, low cost devices themselves promise transformative change in healthcare systems with an increased emphasis on early detection and prevention that lead to positive outcomes, rather than high cost, late stage intervention that often fail.

## Figures and Tables

**Table 1:** Cancer biomarker proteins approved by the US FDA [18].

Biomarker		Sample	Cancer	Clinical Use	Assay
Abbrev.	Name				
Free PSA/fPSa	Free PSA	Serum	Prostate	S, M	Immunoassay
tPSA	Total PSA	Serum	Prostate	S, M	Immunoassay
cPSA	Complex PSA	Serum	Prostate	S, M	Immunoassay
p63	Transformation-related protein 63	FFPE tissue ^†^	Prostate	S, M	Immunohistochemistry
TG	Thyroglobulin	Serum	Thyroid	S, M	Immunoassay
EGFR	Epidermal growth factor receptor	Colon tissue	Colon	Pre	Immunoassay
CEA	Carcinoembryonic antigen	Serum	Colon	M	Immunoassay
MW CEA	High molecular weight CEA	Urine	Bladder	M	Immunofluorescence
FDP (AMDL-ELISA DR-70)	Fibrin/fibrinogen degradation products	Urine/Serum	Bladder	M	Immunoassay
NMP/22	Nuclear matrix protein 22	Urine	Bladder	S, M	Immunoassay
BTA	Bladder tumor antigen	Urine	Bladder	M	Immunoassay
HER2	Human EGF receptor	Serum	Breast	M	Immunohistochemistry
CA15-3 *	Carbohydrate antigen 15-3	Serum, plasma	Breast	M	Immunoassay
CA27-29 *	Carbohydrate antigen 27–29	Serum	Breast	M	Immunoassay
HER/NEU	Human EGF receptor 2	FFPE tissue ^†^	Breast	P, Pre	Immunohistochemistry
ER	Estrogen factor	FFPE tissue ^†^	Breast	P, Pre	Immunohistochemistry
PR	Progesterone factor	FFPE tissue ^†^	Breast	P, Pre	Immunohistochemistry
AFP *	α-fetoprotein	Ser., plasma, amniotic fluid	Testicular	St	Immunoassay
β-hGC *	Human chorionic gonadotropin-β	Serum	Testicular	St	Immunoassay
AFP-L3%	α-fetoprotein L3% isoform	Serum	Hepatocellular	P	HPLC, microfluidic capillary electrophoresis
KIT	Receptor Tyrosine Kinase	FFPE tissue ^†^	Gastrointestinal stromal tumors	Pre	Immunohistochemistry
CA 19-9 *	Carbohydrate antigen 19-9	Serum	Pancreatic	M	Immunoassay
CA 125 *	Carbohydrate antigen 125	Serum	Ovarian	M	Immunoassay
HE4	Human epididymis protein 4	Serum	Ovarian	M	Immunoassay
OVA1 (Multiprotein test	CA125, Apolipoprotein A1, β -2 microglobulin, Transferrin, Pre-albumin	Ovarian	Serum	P	Immunoassay

M—monitoring, S—screening, P—prognosis, Pre—prediction of therapy, St—staging, *—Glycoproteins, FFPE tissue ^†^—formalin-fixed paraffin-embedded (FFPE) tissue slides.

**Table 2:** Clinical characteristics of prostate patient samples.

	Benign	Gleason Score 6	Gleason Score 7	Gleason Score ≥ 8
Number of patients	n = 64 (49%)	n = 3 2 (25%)	n = 22 (17%)	n = 12 (9%)
Age (average, years)	63	67	65	65
Patients with [PSA] ≤ 4.0 ng/mL	50 (78%)	21 (66%)	12 (55%)	4 (33%)
Patients with [PSA] > 4.0 ng/mL	14 (22%)	11 (34%)	10 (45%)	8 (67%)

**Table 3:** Selected 3D-printed biomarker-based cancer diagnostic devices [50,51].

Cancer	Biomarker	Sensor	Range or LOD ^a^
Liver	CD133	Screen-printed gold electrode integrated into a 3D printed chamber	1 × 105 to 3 × 106 HepG2 liver cancer cells/mL [51]
Hepatocellular	Oval cell marker antibody (OV6)	Multiwall carbon nanotube (MWCNT) functionalized electrode integrated into a 3D printed flow cell	1 × 102–5 × 105 hepatic oval cells (HOCs)/mL [52]
Cystic fibrosis	Secretory leukocyte protease inhibitor (SLPI)	Printed circuit board with built-in screen-printed electrode integrated into a 3D printed case and connected to a smart phone for control	Limit of 1 nM [53]
Pancreatic, breast cancer and gastric	Carcinoembryonic antigen (CEA)	Self-designed and printed photoelectrode integrated into a 3D printed platform	10.0 pg/mL–5.0 ng/mL with limit of 4.8 pg/mL [54]
Prostate	Prostate-specific antigen (PSA), prostate-specific membrane antigen (PSMA)	3D printed multiplexed ECL immunoarray with programmable syringe pump	Limits of 150 fg/mL for PSA, and 230 fg/mL for PSMA [55]
Prostate	PSA, cluster of differentiation 14 (CD-14), Golgi membrane protein 1 (GOLM-1), insulin-like growth factor binding protein 3 (IGFBP-3), insulin-like growth factor 1 (IGF-1), platelet factor 4 (PF-4), vascular endothelial growth factor D (VEGF-D), PSMA	3D printed multiplexed ECL immunoarray with lab-built electronic control system	Limits of 78−110 fg/mL [56]
Breast	Nucleolin	Functionalized bipolar electrode (BPE) mounted in a 3D printed microchannel for ECL detection	LOD of 10 MCF-7 breast cancer cells [57]
Prostate	PSA, PS-4	Unibody 3D printed multiplexed CL immunoarray	LOD 0.5 pg/mL [58]
Prostate	PSA, VEGF, IGF-1, CD-14	ELISA based 3D printed multiplexed pipette tip for CL and colorimetric detection	Limits of 5 pg/mL for PSA, 25 pg/mL for VEGF, 2.5 pg/mL for IGF-1, and 0.5 pg/mL for CD-14 [59]
Cervical	Valosin-containing protein (VCP)	Magnetic focus lateral flow immunosensor (mLFS) integrated into a 3D printed frame for colorimetric detection	Limit of 25 fg/mL [60]
Ovarian, breast	VEGF, angiopoietin-2 (Ang-2)	3D-printed immunoarray using lab-formulated carboxyl group rich resin for colorimetric detection	Limit of 11 ng/mL for VEGF, and 0.8 ng/mL for Ang-2 [61]
Oral cancer metastasis	DSG3, VEGF-A, VEGF-C	3D-printed array with cell disruption device to detect metastasis biomarker DSG3 at single cell level	LODs 0.10 fg/mL for DSG3, and 0.20 fg/mL for VEGF-A, VEGF-C and β-Tub [43]

**^a^** Limit of detection (LOD) as signal 3x the average noise + blank; range denotes detectable concentration levels.

## Data Availability

No new data were created or analyzed in this study. Data sharing is not applicable to this article. Original data are available in the referenced articles and their supporting information files found on the respective journal websites.
